# Analysis of the Internal Structure and Psychometric Properties of the Entrapment Scale in Spanish Adolescents and Emerging Adults

**DOI:** 10.3390/ejihpe15060111

**Published:** 2025-06-13

**Authors:** Ana Huertes-del Arco, Eva Izquierdo-Sotorrío, Isabel Ramírez-Uclés, Miguel A. Carrasco, Francisco Pablo Holgado-Tello

**Affiliations:** 1 Department of Personality, Assessment and Psychological Treatments, Faculty of Psychology, Universidad Nacional de Educación a Distancia (UNED), 28040 Madrid, Spain; ahuertes3@alumno.uned.es (A.H.-d.A.); macarrasco@psi.uned.es (M.A.C.); 2 School of Health Sciences and Education, Open University of Madrid (UDIMA), 28400 Madrid, Spain; eva.izquierdo@udima.es; 3 Department of Behavioral Sciences Methodology, Faculty of Psychology, Universidad Nacional de Educación a Distancia (UNED), 28040 Madrid, Spain; pfholgado@psi.uned.es

**Keywords:** suicidal ideation, entrapment scale, adolescents, internal structure, bifactor model

## Abstract

The Entrapment Scale was developed to measure the feeling of being trapped by external situations or internal experiences (such as thoughts and emotions) without the possibility of escape. This perception, especially when combined with feelings of defeat, is central to integrated motivational–volitional (IMV) model of suicidal behavior. This study adapts the Entrapment Scale for Spanish adolescents and emerging adults, focusing on its internal structure, reliability, and criterion validity. We assessed 849 participants (48.1% male) aged 12 to 22 and compared three models: a correlated two-factor model, a second-order factor model, and a bifactor model. The bifactor model showed the best fit, indicating that a general entrapment factor influenced all items, while specific internal and external factors captured unique aspects. Importantly, distinguishing between internal and external entrapment can guide the development of more personalized and effective therapeutic strategies, as the relevance of each dimension may vary depending on the behaviors or symptoms present. This approach allows clinicians to target interventions more precisely to the individual’s needs. Theoretical and practical implications for understanding and addressing suicidal behavior are discussed.

## 1. Introduction

Among the variables contemplated by the second-generation models of suicidal behavior (Yöyen & Keleş, 2024), entrapment plays a fundamental role, as it captures a central psychological subjective experience in the assessment of suicide risk. This construct accounts for the perception of being immobilized in the face of an adverse situation from which there is no possibility of escape. According to the integrated motivational–volitional (IMV; R. C. O’Connor, 2011; R. C. O’Connor & Kirtley, 2018) model, this experience can trigger persistent hopelessness and increase vulnerability to suicide by acting as a key mechanism connecting adverse emotional states with suicidal ideation and behaviors.

Gilbert and Allan’s (1998) Entrapment Scale, which was designed to assess this construct, includes two basic subscales: one for internal entrapment, referring to the experience as derived from internal thoughts and emotions, and one for external entrapment, referring to the experience as derived from external situations or events that may arise at work, in interpersonal relationships, among family, etc., with no perceived possibility of escape. The relevance of this measure lies not only in its ability to identify the subjective experience of entrapment in different contexts and populations but also in its potential to inform preventive intervention strategies—especially among adolescents, for whom the perception of entrapment can be particularly intense and harmful (Huertes-del Arco et al., 2024; Taylor et al., 2011b). This scale takes the entrapment construct from the social rank theoretical model of depression (Gilbert, 1992; J. Price et al., 1994; J. S. Price & Sloman, 1987) and allows it to be used as a salient variable in pain cry theory (Williams, 1997; Williams & Pollock, 2000; Williams, 2001), which links entrapment to experiences of social subordination and chronic stress as well as to the development of emotional problems such as depression and anxiety (Gilbert, 1992; Gilbert & Allan, 1998).

From a theoretical and practical perspective, assessment scales are frequently designed to measure a global psychological trait, such as the experience of psychological entrapment, as well as related subdimensions (e.g., internal and external entrapment in the present case). The items comprising these subscales are developed with the primary objective of detecting relevant behaviors that are both representative of the concepts being evaluated and highly related to each other, according to the theoretical postulates proposed by Slocum-Gori and Zumbo (2011), as well as related (as reflected by their internal consistency) to the global trait (Cronbach, 1951).

In the process of obtaining evidence of the internal structure of a measurement instrument when the overall scores on a scale are reported, it is assumed that the instrument measures a unitary ability or trait and that the scores reflect the level or capacity of the person assessed in terms of that single trait. However, if scores on the trait’s subdimensions are reported, each of these should reflect a sufficiently distinct aspect of the overall construct that is specific to each subdimension (AERA et al., 2014).

Gilbert and Allan’s (1998) Entrapment Scale has been adapted and validated in several cultural and linguistic contexts, producing different results with respect to the structure proposed in the original scale, which was developed as part of a set of three measures designed to assess subjective experiences of defeat and entrapment (Kulemarzi et al., 2024; Martini, 2015; Ordóñez-Carrasco et al., 2021; Tarsafi et al., 2021; Taylor et al., 2009; Trachsel et al., 2010). Within this framework, a specific scale was formulated to measure entrapment, divided into the two dimensions of external entrapment and internal entrapment (Entrapment Scale, Gilbert & Allan, 1998). At the time, it was not clear whether the differentiation between these two types of entrapment presented significant clinical implications or whether a general escape motivation mechanism was more relevant (Gilbert & Allan, 1998).

Previous studies, one with a sample of English university students (Taylor et al., 2009) and another in the German adult population (Trachsel et al., 2010), have shown a unidimensional structure of entrapment with a Cronbach’s alpha coefficient in both studies of 0.95 for the total scale. Kulemarzi et al.’s (2024) study with Iranian adolescents further confirmed that the structure of the Entrapment Scale remains consistent with its original form when applied to adolescent populations. Similarly, Baker et al.’s (2024) research with a military sample supports these findings, in line with previous studies (Cramer et al., 2019; Forkmann et al., 2018). A study by Martini (2015) adapted the Entrapment Scale to a Spanish-speaking adult population, confirming a unidimensional structure; however, Ordóñez-Carrasco et al. (2021), also studying a Spanish adult population, found a compatible dimensionality with both a two-dimensional and a unidimensional structure and internal consistency values above 0.95 for the total scale (i.e., including both internal and external entrapment). It should be noted that in works with Spanish-speaking samples, the different versions of the scale have exhibited adequate semantic and conceptual equivalence with the original instrument and psychometric properties comparable to those reported by the original authors (Gilbert & Allan, 1998).

Research measuring entrapment has shown good reliability, with Cronbach’s alpha values for the scales and subscales ranging from 0.86 to 0.96. There is also evidence of adequate convergent validity with theoretically related constructs such as anxiety, depression, hopelessness, defeat, and suicidal behaviors; in addition, evidence of criterion validity has been reported in relation to these variables (e.g., Gilbert & Allan, 1998; Panagioti et al., 2013; Taylor et al., 2011a; Thompson & Swartout, 2018; Willner & Goldstein, 2001).

The study of entrapment during adolescence is particularly important given the developmental characteristics of this life stage and the high prevalence of suicidal thoughts and behaviors (STBs) (Asarnow & Ougrin, 2019; Cha et al., 2018; Glenn et al., 2020). Adolescence and emerging adulthood are marked by profound psychosocial transitions, identity formation, autonomy, and vocational decision making, all while the neurocognitive systems responsible for emotional regulation and executive functioning are still maturing (Arnett, 2000; Casey et al., 2011; Schulenberg et al., 2004). This developmental mismatch often increases vulnerability to internalizing symptoms such as depression and suicidal ideation, as evidenced by elevated rates of self-injury and suicide risk in youth populations (Cha et al., 2018; Glenn et al., 2020; Kessler et al., 2005; Nock et al., 2013). The entrapment construct has been empirically linked to psychological maladjustment, particularly depressive symptoms and suicidal behaviors (Taylor et al., 2011a, 2011b; Galynker et al., 2017; Huertes-del Arco et al., 2024; Littlewood et al., 2016; R. C. O’Connor & Kirtley, 2018; D. B. O’Connor et al., 2021). Recent evidence (Van Eijk et al., 2023) further underscores entrapment as a key variable in distinguishing levels of suicide risk in the general population and, more notably, within clinical and high-risk groups (R. C. O’Connor et al., 2013; Branley-Bell et al., 2019), reinforcing its central role in contemporary theoretical models of suicidal behavior (R. C. O’Connor & Kirtley, 2018). In this context, we present evidence on the psychometric properties of an entrapment scale administered to a sample of Spanish adolescents and emerging adults, thereby contributing to its validation and utility in applied settings. Given the recognized need for cross-cultural research into the psychological correlates of entrapment (R. C. O’Connor & Portzky, 2018), particularly across sociodemographic groups, this study also aligns with epidemiological and theoretical findings emphasizing age, gender, and cultural variations in STBs and their associated risk and protective factors (Borges et al., 2010; Cramer et al., 2019; Davidson & Wingate, 2011; Drapeau & McIntosh, 2017; Keyes & Platt, 2024; Klonsky & May, 2014; Wolford-Clevenger et al., 2018). Finally, the ongoing validation of the scale, including evidence from internal structure and criterion validity, aims to support both research and clinical assessment efforts (Iliescu et al., 2024; Markus & Borsboom, 2024).

The aim of the present study is to analyze some basic psychometric properties of the adapted version of Gilbert and Allan’s (1998) Entrapment Scale as administered to a sample of Spanish adolescents and young adults. Three different models are explored here: (a) a model with two correlated factors (internal and external entrapment); (b) a model in which both entrapment factors (internal and external) are explained by a single second-order factor (general entrapment); and (c) a bifactor model where the two entrapment factors (internal and external) coexist as specific factors, while a general factor captures the shared variance of the set of items. Additionally, we analyze reliability and criterion validity.

## 2. Materials and Methods

### 2.1. Participants

After reviewing the database, 49 participants with more than 10% missing values were eliminated (Cohen & Cohen, 1983). The remaining participants were treated with pairwise deletion; this optimizes the sample size for analyses that use correlation or covariance matrices as input because each pair of variables is evaluated based only on participants with non-missing values for each pair of variables. The sample ultimately comprised 849 individuals between 12 and 22 years of age (*M* = 15.43, *SD* = 2.26) who were students in educational centers in Spain (51% female, 0.7% non-binary, and 0.2% who did not respond to the question).

### 2.2. Procedure

In accordance with the guidelines proposed by the International Test Commission (ITC) (2010) for test adaptation and translation, a rigorous back-translation process was carried out. Initially, two independent experts separately translated the test into the target language, ensuring that as much semantic, conceptual, and cultural equivalence was maintained as possible. Subsequently, a third person with expertise in the subject matter compared the two versions, resolving discrepancies and ensuring fidelity to the original content. This strategy minimized possible biases and ensured that the translated version was applicable in the new linguistic and cultural context.

Data collection was carried out collectively in multiple secondary schools, vocational training schools, and universities by means of online forms. The process was assisted by trained evaluators. All participants gave their informed consent, as did their respective schools. In the case of minors under 16 years of age, the informed consent of their legal guardians was also obtained.

The study was approved by the university ethics committee (Ref. (UNED) [12-PSI-2022]).

### 2.3. Instruments

The Entrapment Scale developed by Gilbert and Allan (1998) was designed to assess the subjective perception of being trapped in adverse situations with no possibility of escape (research version translation and adaptation: Grupo Investigación Psicológica en Población Infanto-Juvenil, Universidad Nacional de Educación a Distancia; based on the original scale by Gilbert & Allan, 1998). The original scale consists of 16 items, divided into two subscales. Internal entrapment (six items) assesses the feeling of being trapped inside oneself due to negative thoughts and emotions, such as self-criticism or hopelessness (e.g., “I want to escape from myself”); External entrapment (10 items) measures the perception of being trapped in external circumstances or unfavorable social situations that cannot be modified, with no possibility of escape (e.g., “I am in a situation in which I feel trapped”). Each item is scored on a 5-point Likert scale from 0 (“Does not describe me at all”) to 4 (“Describes me completely”). The total score, obtained by summing the responses of all items, can range from 0 to 64 points. The original authors indicate that higher scores show a higher level of entrapment, but they do not establish cutoff points to classify the subjects. The following were obtained for the present sample: a Cronbach’s alpha of 0.95 and a McDonald’s omega of 0.96 for general entrapment, a Cronbach’s alpha of 0.93 and a McDonald’s omega of 0.93 for the internal entrapment subscale, and a Cronbach’s alpha of 0.93 and a McDonald’s omega of 0.92 for the external entrapment subscale.

Previous studies have shown good psychometric properties, with Cronbach’s alpha coefficients generally above 0.85 for both the total scale and the subscales (Cramer et al., 2019; Forkmann et al., 2018; Gilbert & Allan, 1998; Martini, 2015; Ordóñez-Carrasco et al., 2021; Taylor et al., 2009; Trachsel et al., 2010). The original subscales proposed by Gilbert and Allan (1998) showed good internal consistency, both in a group of university students (Cronbach’s alpha of 0.93 for the internal entrapment scale and 0.88 for the external one) and in a sample of people with depression (Cronbach’s alpha of 0.86 for the internal entrapment scale and 0.89 for the external one). In a study conducted in Germany, the Cronbach’s alpha of the scale was 0.95 for both a sample that completed the questionnaire in person and a sample that completed it online (Trachsel et al., 2010). In the study conducted in Spain by Ordóñez-Carrasco et al. (2021) with an adult population, the Cronbach’s alpha coefficient was 0.96, the split-half reliability coefficient was 0.98, and the McDonald’s omega coefficient was 0.97.

The Defeat Scale (research version translation and adaptation: Grupo Investigación Psicológica en Población Infanto-Juvenil, Universidad Nacional de Educación a Distancia; based on the original scale by Gilbert & Allan, 1998) is a 16-item self-administered scale. It assesses the feeling of defeat, referring to the perception of failed struggle or failure as a result of experiences of rejection, loss, and/or significant alteration of social status, identity, or goal attainment during the past week (e.g., “I feel that I have failed in life”). It is presented in a 5-point Likert-type format from 0 (“never”) to 5 (“always”). Items 2, 4, and 9 are reversed and refer to perceiving oneself as a winner or successful person. Possible total scores on the defeat scale range from 0 to 64 points; the higher the score, the higher the perception of being defeated. The original scale, understood as unidimensional, showed good internal consistency in a sample of students with a Cronbach’s alpha of 0.94 and with a Cronbach’s alpha of 0.93 in a sample of people with depression (Gilbert & Allan, 1998). In the present sample, it obtained a Cronbach’s alpha of 0.94 and a McDonald’s omega of 0.94.

The Beck Hopelessness Scale (Beck et al., 1974; Beck & Steer, 1993), Spanish version (Hermosillo-De la Torre et al., 2020; Robi, 2012; Tovar, 2011), is a 20-item self-report scale that assesses the degree to which the respondent’s expectations of the future are negative as well as their ability to cope with difficulties in life (e.g., “I look forward to the future with hope and enthusiasm” or “I could give up since I cannot do things better on my own”). Each item is scored on a 5-point Likert scale ranging from 0 (“Does not fit my reality at all”) to 4 (“Completely fits my reality”). Total possible scores range from 0 to 100 points; the higher the score, the greater the feeling of hopelessness. Spanish adaptations of this scale have shown good psychometric properties, with Cronbach’s alphas of between 0.70 and 0.75 (Aguilar et al., 1995; Viñas-Poch et al., 2004). In the present sample, it obtained a Cronbach’s alpha of 0.79 and a McDonald’s omega of 0.80.

The Child and Adolescent Assessment System (SENA; Fernández-Pinto et al., 2015) is a self-report scale that assesses a wide range of emotional and behavioral problems in children and adolescents. The items are presented in Likert format, with response options ranging from 1 to 5, from “never or almost never” to “always or almost always.” In the present study, the Depression Scale (14 items; e.g., “I feel that no one cares about what I do” or “I think my life has no meaning”) was used exclusively. This scale has shown good psychometric properties, with Cronbach’s alphas between 0.70 and 0.87 (Flores et al., 2022; Sánchez-Sánchez et al., 2016). In the present sample, the Depression Scale obtained a Cronbach’s alpha of 0.92 and a McDonald’s omega of 0.83.

The Interpersonal Needs Questionnaire (INQ-12; Van Orden et al., 2012) is a 15-item self-report scale that assesses the perception of “feeling like a burden” and a feeling that has been dubbed “thwarted belongingness”. The former refers to the sense of being a burden to oneself, friends, family, or society (e.g., “Those around me would be better off if I were gone”); the latter, “thwarted belongingness”, refers to the subjective experience of feeling lonely or disconnected from friends, family, or other valued social circles (e.g., “I feel close to other people”). Both dimensions are assessed on a 7-point Likert-type scale, ranging from 1 (“Not at all”) to 7 (“Extremely”). Items 7, 8, 10, 13, 14, and 15 are reversed. The instrument has shown a significant association with suicidal ideation in the general population. Both subscales have shown adequate internal consistency, with Cronbach’s alphas between 0.92 and 0.95 for “feeling like a burden” and 0.80 for “thwarted belongingness” (Ordóñez-Carrasco et al., 2018, 2021). In the present sample, a Cronbach’s alpha of 0.95 and a McDonald’s omega of 0.95 were obtained for the “feeling like a burden” subscale; a Cronbach’s alpha of 0.83 and a McDonald’s omega of 0.82 were obtained for the “thwarted belongingness” subscale.

### 2.4. Data Analysis

The data analysis included a study of the descriptive characteristics of the sample and an examination by confirmatory factor analysis (CFA) of the original structure proposed by Gilbert and Allan (1998), with its two dimensions of internal and external entrapment. The original structure can be analyzed from the standpoint of three different models. The first is the correlated factor model, which allows the test’s multidimensionality to be captured explicitly. Unlike hierarchical or bifactor models, the correlated factor model does not include an underlying general factor that explains the covariation between dimensions. Instead, the correlations between the latent variables represent their shared variability.

Two alternative models were also tested: second-order factor and bifactor models. The second-order factor model represents a hierarchical extension of the correlated factor model. In this model, the latent first-order factors are grouped under the influence of a single second-order factor that explains the covariation between them. This approach makes it possible to model a structure in which a general dimension influences all the specific dimensions but without directly affecting the observed variables, thus providing a more parsimonious structural representation at scales where the latent factors are highly intercorrelated. This hierarchical model thus facilitates the interpretation of the factorial structure by distinguishing between a global component and specific dimensions, which is appropriate in the evaluation of constructs with multiple interrelated facets.

Finally, the bifactor model (Holzinger & Swineford, 1937), also called the nested factor model (Brunner et al., 2012; Gustafsson & Åberg-Bengtsson, 2010) or hierarchical model (Markon, 2019), incorporates not only a general factor that loads directly on all observed variables but also grouping factors that affect subsets of these variables. A distinguishing feature of this model is that the general factor and the grouping factors are orthogonal, i.e., they do not share variance. Unlike traditional confirmatory factor models, the bifactor model does not have a simple structure in which each observed variable is only associated with a single factor (Gustafsson & Åberg-Bengtsson, 2010). Instead, each variable loads on both the general factor and a specific grouping factor, implying that the explained variance is split between at least two sources. This feature is important in differentiating it from the second-order factor model, in which the general factor exerts its influence indirectly through the first-order factors. In the bifactor model, in contrast, the relationship with the general factor is direct, giving the latter greater theoretical weight (Markon, 2019).

Given the characteristics of the data, statistical analyses were based on the polychoric correlation matrix, and robust unweighted least squares (RULS) was used as the estimation method. When the variables measured are ordinal, the Pearson correlation tends to underestimate the association between them. An undesirable consequence is bias in the factor loadings obtained (Holgado-Tello et al., 2010). However, according to Jöreskog and Sörbom (1996), polychoric correlation is the most robust estimator. Additionally, when ordinal variables are used, RULS presents fewer type I errors compared to methods as maximum likelihood (ML), robust maximum likelihood (RML), weighted least squares (WLS), and unweighted least squares (ULS) (Holgado-Tello et al., 2018). To evaluate the goodness-of-fit of the models, the chi-square statistic (χ^2^) was calculated, together with the following fit indices: standardized root mean squared residuals (SRMR), root mean squared error of approximation (RMSEA), comparative fit index (CFI), non-normed fit index (NNFI), adjusted goodness-of-fit index (AGFI), normed fit index (NFI), and incremental fit index (IFI). The interpretation of the fit indices was based on the criteria proposed by Hu and Bentler (1999). The reliability of the scores was assessed using Cronbach’s alpha (Zumbo & Rupp, 2004) and McDonald’s omega (Ventura-León & Caycho-Rodríguez, 2017) coefficients. Finally, to explore the evidence of criterion validity based on relationships with other variables, bivariate and partial correlation analyses were applied to the relationships between the entrapment scores (internal and external), and the rest of the scores obtained through the other instruments used in the study.

The statistical programs used were SPSS version 20.0 for Windows and LISREL 8.80.

The statistical programs employed in this study are functionally equivalent in both the versions used and the current ones.

## 3. Results

### 3.1. Descriptive Item Analysis

Table 1 shows a descriptive analysis of the scores on the entrapment scale items, including the means, standard deviations, and skewness and kurtosis coefficients.

### 3.2. Confirmatory Analysis

In the confirmatory factor analysis, three different models were contrasted for this scale: (a) a correlated factor model with two factors, (b) a second-order factor model, and (c) a bifactor model. The two correlated factors correspond to the original model of Gilbert and Allan (1998); the aim of the second-order factor model is to obtain evidence for the existence of a general factor called “entrapment”, in which the two specific factors of internal and external entrapment are nested. Finally, with the bifactor model, the aim is to determine whether the test can be considered sufficiently unidimensional for only one score to be obtained and whether it would also make sense to obtain scores on each of the subscales.

The completely standardized solution for the three examined models is shown in Table 2.

The second-order factor model has a chi-square (χ^2^) of 835.95 with 104 degrees of freedom, indicating an overall model fit. The RMSEA of 0.05 and SRMR of 0.08 suggest an adequate fit. The CFI of 0.93 and AGFI of 0.96 indicate an acceptable fit. The NNFI of 0.92 and IFI of 0.93 both reflect a moderate fit. The correlated factor model has a chi-square (χ^2^) of 609.49 with 103 degrees of freedom. The RMSEA of 0.05 and SRMR of 0.05 indicate a good fit. The CFI of 0.95 and AGFI of 0.99 also show a good fit. The NNFI of 0.95 and IFI of 0.95 both indicate a good model fit. Finally, the bifactor model has a chi-square (χ^2^) of 429.73 with 88 degrees of freedom. The RMSEA of 0.05 and SRMR of 0.03 indicate an excellent fit. The CFI of 0.97 and AGFI of 0.99 both suggest a very good fit. The NNFI of 0.96 and IFI of 0.97 both reflect an excellent model fit.

As shown in Table 3, the three evaluated models all show an adequate fit according to the goodness-of-fit indices. However, the bifactor model demonstrates the best overall fit, with exceptionally high values for the incremental fit indices, such as the CFI (0.97), NNFI (0.96), and IFI (0.97) as well as an exceptionally low SRMR (0.03). The correlated factor model also shows a good fit, with a CFI of 0.95 and an SRMR of 0.05, indicating that it is a solid option. Lastly, the second-order factor model, while still acceptable, shows the lowest values compared to the other two models, with a CFI of 0.93 and an SRMR of 0.08. In summary, the bifactor model shows the better fit across all key indices.

First, the observed results demonstrate that the fit indices for the correlated factor model show acceptable values, indicating that this model captures a coherent two-dimensional structure. Second, the second-order factor model, which postulates a global factor that indirectly influences the items through the two specific factors, also shows an adequate fit. This could be interpreted as evidence that although there are two distinct dimensions, both respond to a higher latent construct.

Finally, at a purely descriptive level, the bifactor model presents adequate and slightly—if minimally—superior fit indices. This model suggests the presence of a general factor that explains most of the variance shared by all the items, while the specific factors of internal and external entrapment explain independent variances not captured in general entrapment (GE), specifically represented by the items 1, 3, and 4 for IE and the elements 8, 9, 10, 15, and 16 for EE.

Table 3 shows that the mean saturation for internal entrapment is 0.83 for the second-order factor model, while for external entrapment, it is 0.73. These relatively high values suggest that both factors have considerable weight in explaining the variance of the overall construct, indicating a balanced contribution of both dimensions to the model.

In the correlated factor model, slightly lower means (0.82 for internal entrapment and 0.72 for external entrapment) show a similar trend but with a lower relative contribution, possibly due to the direct correlation between the two factors.

Finally, the bifactor model reveals a significant finding: while the general entrapment factor (mean loading = 0.75) acts as a unifying core, the internal and external dimensions (mean loadings = 0.21 each) function as complementary nuances. Imagine the concept of entrapment as the central theme in a story. The main plot revolves around this overarching idea (the general factor), while the subplots (specific factors) add depth to specific characters (internal vs. external experiences). Statistically, this means that the shared experience of feeling trapped dominates the scale’s variance, while the distinction between internal and external distinctions provides finer-grained information.

This pattern aligns with the theoretical view of entrapment as a unified construct with two secondary dimensions. For example, an item like “I would like to escape from my thoughts and feelings” might primarily reflect the general factor (strong loading = 0.79) but still carry a small “signal” specific to internal entrapment (loading = 0.27). These lower specific loadings do not negate the importance of distinguishing between internal and external entrapment; rather, they highlight that these dimensions are best interpreted alongside the general factor.

Given the results obtained and the comparison of the three models under analysis, we can assume that the use of two specific scores (internal and external entrapment) along with a general score (general entrapment) is theoretically sound and psychometrically feasible, offering a more robust tool for analysis and practical interventions. From the applied perspective, in clinical contexts, these specific scores may be useful for identifying individual differences in the internal and external dimensions of entrapment, guiding differentiated therapeutic strategies. For example, a high score for internal entrapment might reflect self-critical and ruminative cognitive processes, whereas a high score for external entrapment might be more related to adverse social or environmental circumstances. The overall score, on the other hand, provides an integrated measure of the overall level of entrapment by offering a measure of global severity.

**Figure 1:**
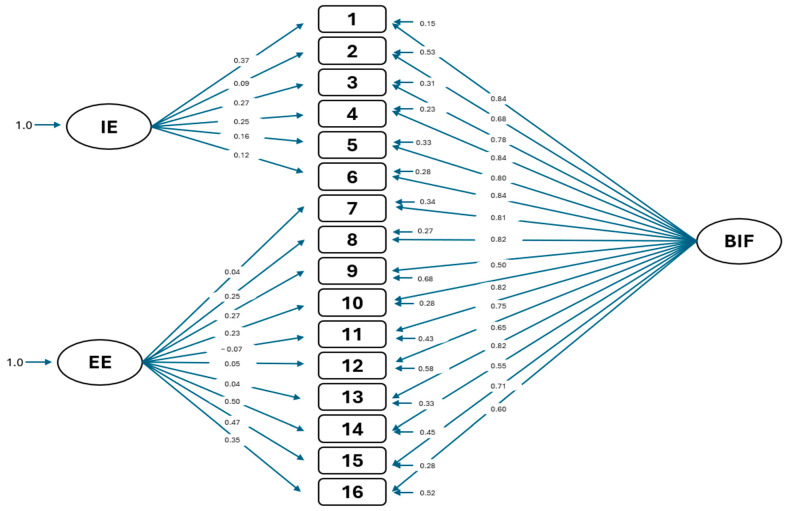
Bifactor model.

Figure 1 shows the bifactor model of the Entrapment Scale.

### 3.3. Reliability

To assess the internal consistency of the study’s measures, we calculated McDonald’s omega (ω) and Cronbach’s alpha (α) coefficients. Both indicators provide estimates of scale reliability, with values above 0.70 generally considered acceptable and those above 0.90 indicating excellent reliability. As shown in Table 4, general entrapment exhibited excellent internal consistency, with ω = 0.96 and α = 0.95, indicating a high level of reliability. Similarly, the internal entrapment subscale obtained ω = 0.93 and α = 0.93, while the external entrapment subscale showed ω = 0.93 and α = 0.92. These values suggest that both subscales also demonstrate excellent internal consistency. Overall, the reliability indices indicate that both the general scale and its subscales provide consistent and reliable measurements, supporting their use in further analyses.

### 3.4. Criterion Validity

To analyze the criterion validity, we calculated the Pearson bivariate correlation between the subscales of entrapment (external and internal) and the scores on (a) perception of defeat, (b) severity of depressive symptomatology, (c) hopelessness, (d) thwarted belongingness, and (e) the feeling of being a burden. Given the interrelation between the entrapment scales, we also obtained the partial correlation between each subscale and each of the above-mentioned measures, controlling in each case for the effect of the remaining subscale (Table 5).

The results indicate that both total and partial correlations are positive and significant across all variables. Internal entrapment shows strong total correlations with defeat (0.78) and depression (0.79) and moderate to high correlations with thwarted belongingness (0.51), hopelessness (0.60), and perceived burdensomeness (0.66). This means that, in general, feeling trapped by one’s own thoughts and emotions is closely linked to feeling defeated, depressed, isolated, hopeless, and burdensome.

However, when we look at partial correlations—that is, when we control for the effect of external entrapment—we see that these relationships become notably weaker. For example, the correlation between internal entrapment and thwarted belongingness drops sharply from 0.51 to just 0.08. This suggests that the connection between feeling like one do not belong and feeling internally trapped is mostly explained by the general experience of entrapment or by external factors rather than being something unique to internal entrapment itself. In other words, once we account for the broader sense of being trapped (including external circumstances), the specific link between thwarted belongingness and internal entrapment almost disappears.

In contrast, for external entrapment, even after controlling for internal entrapment, the partial correlation with thwarted belongingness remains relatively strong (0.29). This indicates that feeling excluded or disconnected from others is more specifically and uniquely related to feeling trapped by external situations—such as social or environmental barriers—than to internal struggles alone.

A similar pattern is observable for other variables: the partial correlations for internal entrapment drop more sharply than those for external entrapment, meaning that external entrapment maintains a more independent and robust relationship with these psychological variables.

To summarize, while both internal and external entrapment are linked to negative psychological states, external entrapment appears to have a more consistent and unique association with feelings of social exclusion, defeat, depression, and burdensomeness, even when internal entrapment is taken into account. This distinction is important for clinical practice, as it suggests that interventions targeting social connection and external circumstances may be particularly helpful for those experiencing external entrapment.

## 4. Discussion

The present study aims to provide empirical evidence on the internal structure and core psychometric properties of the Entrapment Scale in a Spanish adolescent population. While Gilbert and Allan (1998) originally conceptualized entrapment as a two-dimensional construct with internal and external dimensions, subsequent studies (Ordóñez-Carrasco et al., 2021; Taylor et al., 2009; Trachsel et al., 2010) have highlighted the strong correlation between the two dimensions, supporting a unidimensional interpretation. Nevertheless, in the current study, a bifactor model was also tested and found to provide the best statistical fit.

To date and to the best of our knowledge, although the dimensionality of entrapment has been previously explored, few studies have focused on comparing different dimensionality models to identify the model that best fits the adolescent population, specifically for Spanish adolescents. In this sense, the present study, beyond analyzing the dimensionality of the scale, offers a comparison between different dimensional models as applied specifically to the Spanish adolescent population. According to these results, the presence of a general factor is responsible for explaining most of the variance shared by all the items, while the specific factors of internal and external entrapment are responsible for explaining the independent residual variances, represented by items such as “*I want to get away from myself*” (item 1) for IE or “*I have a strong desire to get away and stay away from where I am now*” (item 9) for EE.

From an applied perspective, this finding reinforces the idea that the overall feeling of entrapment could be the core factor underlying the experience of entrapment, while the internal and external dimensions reflect nuances about the specific sources of that feeling. From an applied point of view, consideration of any other construct (e.g., depression, hopelessness, and suicidal behavior) that is associated with entrapment should take into account both the main underlying core and the specific dimensions of the measure in question. This will facilitate more precise understanding of the relevant relationships.

Although the integrated motivational–volitional (IMV) model of suicidal behavior does not distinguish between internal and external entrapment (R. C. O’Connor, 2011; R. C. O’Connor & Kirtley, 2018), and many early studies treated these constructs as overlapping due to their conceptual proximity (Taylor et al., 2011a), recent empirical findings underscore the importance of differentiating them. Carvalho et al. (2013) confirmed that both internal and external entrapment correlate with perceived defeat, consistent with Gilbert and Allan’s (1998) foundational work. However, growing evidence suggests that internal entrapment plays a more salient role in suicidal ideation. For instance, studies by De Beurs et al. (2019), Lucht et al. (2020), and Owen et al. (2018) demonstrated a stronger association between suicidal ideation and internal entrapment than suicidal ideation and external entrapment. Similarly, Rasmussen et al. (2010) identified internal entrapment as the key mediator in the transition from defeat to suicidal ideation, highlighting its relative resistance to change and its critical role in suicide risk. Complementing this, Forkmann et al. (2018) employed network analysis to argue for the necessity of parsing subdimensions of entrapment, particularly given the limitations of traditional factor analysis in highly interrelated constructs (Griffiths et al., 2015). Longitudinal data from psychiatric samples further supports this distinction: Höller et al. (2022) found that only internal entrapment was significantly associated with suicidal ideation over short time frames, a finding that converges with those of previous studies (e.g., Rasmussen et al., 2010; Owen et al., 2018). Most recently, a systematic review by Souza et al. (2024) confirmed that internal entrapment consistently mediates the defeat–suicidal ideation link in both cross-sectional and longitudinal designs, whereas external entrapment demonstrates limited or inconsistent effects. Nonetheless, current evidence remains heterogeneous, and there is a pressing need for prospective, cross-population studies specifically investigating the distinct contributions of internal versus external entrapment to suicidal ideation.

Regarding evidence of criterion validity based on relationships with other variables, the results are consistent with the findings obtained in previous works. Specifically, the high, positive, and statistically significant relationship between depressive symptomatology, defeat, and entrapment is consistent with the results of several studies (Carvalho et al., 2013; Huertes-del Arco et al., 2024; Ordóñez-Carrasco et al., 2021; Panagioti et al., 2017). Regarding the relationships between thwarted belongingness, the feeling of being a burden, and entrapment, the results are consistent with those of previous studies (Dhingra et al., 2016; Forkmann & Teismann, 2017; Ordóñez-Carrasco et al., 2021). In addition, in our study, thwarted belongingness shows relevant relationships with both internal and external entrapment but a stronger one with external entrapment. The feeling of being a burden, which shows a stronger relationship than thwarted belongingness with both internal and external entrapment, is also more closely associated with external entrapment. As these nuances illustrate, analyzing the differences between the internal and external subdimensions of entrapment and how each relates specifically to other variables of interest could be relevant for defining intervention targets. Finally, the positive relationship between entrapment and hopelessness is notable, as pointed out by the works of Panagioti et al. (2012, 2013) and the present study, particularly in the case of internal entrapment.

These results suggest that the entrapment scale presents favorable validity evidence regarding its relationship with the key variables associated with suicidal behavior, such as defeat, depression, hopelessness, thwarted belongingness, and the feeling of being a burden. However, future studies should investigate these relationships through network analysis.

The results of this work suggest the importance of selecting specific intervention targets in relation to the experience of entrapment. According to the bifactor structure, psychological interventions should focus primarily on reducing the overall feeling of entrapment by addressing the specific variables that distinguish the internal sources (such as self-critical thoughts, rumination, and persistent hopelessness) from the external sources (social circumstances perceived as inescapable) responsible for the more global feeling of entrapment. The distinction between the two dimensions will matter in proportion to the relevance of each dimension to the various behaviors requiring intervention, particularly in relation to the most representative items of each type of entrapment. Differentiating between internal and external entrapment can facilitate the development of more personalized and effective therapeutic strategies tailored to the specific type of entrapment experienced by the individual. In addition, this differentiation may enhance patients’ insight into their own suicidality, as each form of entrapment may serve as a contributing factor to suicidal thoughts or behaviors (Cramer et al., 2019; Tucker et al., 2015).

An important aspect of these results to be analyzed in future work relates to the interpretation of the scores and the establishment of criteria to classify subjects according to the levels of the various entrapment-related traits that they display.

This study has certain limitations that should be considered when interpreting the results. First, the use of incidental sampling restricts the generalizability of the findings to other populations. Likewise, the sample size used, although relatively large, did not allow for the creation of independent subsamples within which different patterns could be explored. Furthermore, the cross-sectional nature of the study did not allow us to analyze the stability of the measures. Finally, since all the measures were obtained through self-reporting, a portion of the relationships between the different variables may be explainable by the effects of the method and the informant sourcing.

Despite these limitations, the present study contributes some evidence of construct validity in the Spanish adolescent and juvenile population. This allows the use of the entrapment scales in this cultural setting and opens up new avenues of exploration in Spanish samples with specific clinical or psychosocial conditions. The results of this study offer an alternative conceptual framework for understanding the possible role of entrapment in relation to other constructs and identifying specific intervention strategies that reflect the relevance (or relative weight) of internal versus external entrapment in each case.

Future research should replicate this study in different, larger samples with specific clinical and psychosocial characteristics to determine the extent of entrapment’s role in explaining different case histories and to support the use of entrapment scales and their implications. Likewise, assessing factorial invariance as a function of other moderators such as gender or age will permit examination of the differential behavior of items and scales, which would contribute to a deeper understanding of the validity and applicability of this scale.

Additionally, future research would greatly benefit from utilizing longitudinal designs or ecological momentary assessment (EMA) methodologies (Kleiman et al., 2017) to better capture the temporal dynamics and fluctuations of entrapment, especially in relation to changeable suicidal behavior (Glenn et al., 2022; Kivelä et al., 2024). These approaches would enable researchers to monitor changes in entrapment over time and in relation to specific life events or daily stressors, thereby providing a deeper and more nuanced understanding of its role in the transition from suicidal ideation to action. Employing such methodologies could also help to identify critical periods or contexts in which interventions may be most effective (Lucht et al., 2020; Kraiss et al., 2024), ultimately enhancing the clinical relevance and applicability of the entrapment construct (Davidson et al., 2016).

In conclusion, the present study shows that the bifactor model not only offers the best fit but also provides an alternative conceptual framework for understanding the role of entrapment in suicidal behavior, which is conducive to a more tailored clinical approach adapted to individual needs.

## Figures and Tables

**Table 1:** Descriptive statistics for the item scores.

Items	*M*	*SD*	Skewness	Kurtosis
Item 1	1.70	1.07	1.22	0.61
Item 2	1.97	1.16	0.69	−0.56
Item 3	2.24	1.36	0.61	−0.79
Item 4	1.62	1.10	1.50	1.49
Item 5	1.80	1.27	1.26	0.49
Item 6	1.54	1.06	1.69	2.22
Item 7	1.61	1.02	1.51	1.50
Item 8	1.41	0.90	2.14	4.20
Item 9	1.30	0.78	2.62	7.34
Item 10	1.68	1.11	1.44	1.13
Item 11	1.87	1.14	0.99	0.32
Item 12	2.14	1.22	0.74	−0.33
Item 13	1.63	1.03	1.41	1.18
Item 14	1.31	0.79	2.60	7.20
Item 15	1.57	1.06	1.75	2.30
Item 16	1.41	0.91	2.22	4.63

Note. *M* = mean; *SD* = standard deviation. Items adapted to Spanish from the original version.

**Table 2:** Completely standardized solution for the three models.

Item	Correlated Factor Model		Second-Order Factor Model (GE)		Bifactor Model		
	IE	EE	IE	EE	IE	EE	Bifactor
1	0.89	---	0.74	---	0.37	---	0.84
2	0.69	---	0.71	---	0.09	---	0.68
3	0.82	---	0.84	---	0.27	---	0.79
4	0.88	---	0.90	---	0.25	---	0.84
5	0.82	---	0.85	---	0.16	---	0.80
6	0.86	---	0.88	---	0.12	---	0.84
7	---	0.82	---	0.63	---	0.04	0.81
8	---	0.85	---	0.87	---	0.25	0.82
9	---	0.53	---	0.54	---	0.27	0.49
10	---	0.85	---	0.87	---	0.23	0.82
11	---	0.74	---	0.76	---	−0.07	0.75
12	---	0.66	---	0.67	---	0.05	0.65
13	---	0.82	---	0.84	---	0.04	0.82
14	---	0.60	---	0.62	---	0.5	0.55
15	---	0.75	---	0.77	---	0.47	0.71
16	---	0.64	---	0.65	---	0.35	0.60
IE	---	---	---	---	---	---	---
EE	0.94	---	---	---	---	---	---
GE	---	---	0.92	0.97	---	---	---

Note. IE = internal entrapment; EE = external entrapment; GE = general entrapment.

**Table 3:** Fit measures for the correlated factor model, the second-order factor model, and the bifactor model with average loadings.

Fit Index	Correlated Factor Model	Second-Order Factor Model	Bifactor Model
Chi-square (χ^2^)	609.49 (103)	835.95 (104)	429.73 (88)
RMSEA	0.05	0.05	0.05
SRMR	0.05	0.08	0.03
CFI	0.95	0.93	0.97
AGFI	0.99	0.96	0.99
**Mean (SD) of factor loading**			
Internal entrapment	0.82 (0.07)	0.83 (0.07)	0.21 (0.10)
External entrapment	0.72 (0.11)	0.73 (0.11)	0.21 (0.18)
Bifactor	--	--	0.75 (0.11)

**Table 4:** Descriptive statistics and reliability.

	Mean	SD	McDonald’s ω	Cronbach’s α	Mean Discrimination
General entrapment	26.81	13.26	0.96	0.95	0.73
Internal entrapment	10.86	6.07	0.93	0.93	0.79
External entrapment	15.94	7.75	0.93	0.92	0.70

Note: SD = standard deviation.

**Table 5:** Pearson correlations (*r*) and partial correlations (*pr*) between the entrapment dimensions (internal and external) and the measures of defeat, thwarted belongingness, perceived burdensomeness, depression, and hopelessness.

	Internal Entrapment (*r*)	External Entrapment *(r*)	Internal Entrapment (*pr*)	External Entrapment (*pr*)
Defeat	0.78 **	0.79 **	0.35 **	0.41 **
TB	0.51 **	0.57 **	0.08 *	0.29 **
PB	0.66 **	0.71 **	0.18 **	0.37 **
Depression	0.79 **	0.80 **	0.36 **	0.43 **
Hopelessness	0.60 **	0.56 **	0.28 **	0.14 **

Note. TB = thwarted belongingness; PB = perceived burdensomeness; (*r*) = Pearson correlation; (*pr*) = partial correlation. ** *p* < 0.001; * *p* < 0.01.

## Data Availability

The data that support the findings of this study are available from the corresponding author upon reasonable request, in compliance with ethical guidelines and participant confidentiality.
